# Supplementary figures and images for: Deep learning-based approach to the characterization and quantification of histopathology in mouse models of colitis
Source: PLoS One. 2022 Aug 29;17(8):e0268954. doi: 10.1371/journal.pone.0268954 (PMC9423669; doi:10.1371/journal.pone.0268954)

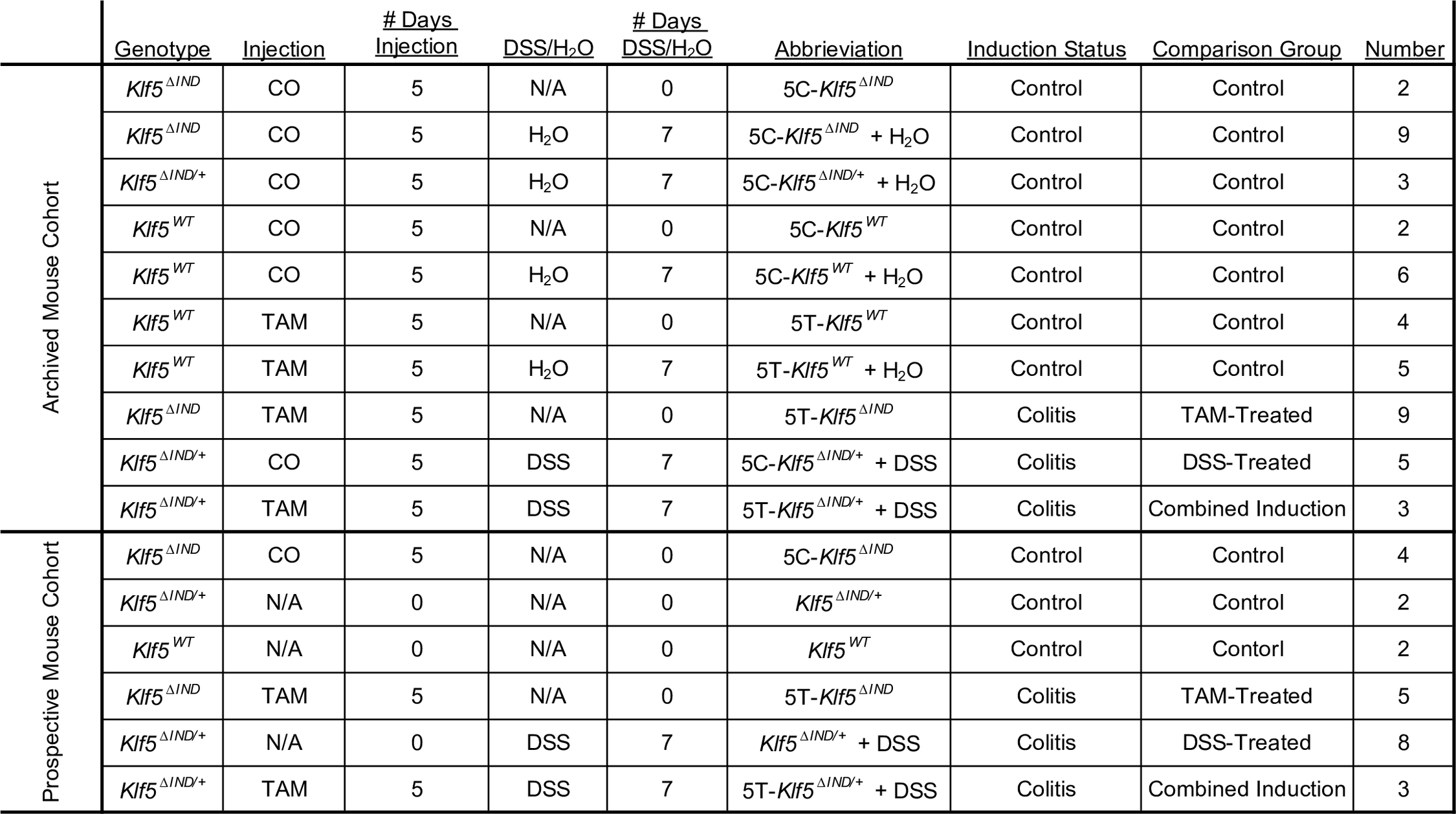

Supplement: S1 Table — (TIF) [file pone.0268954.s001.tif]

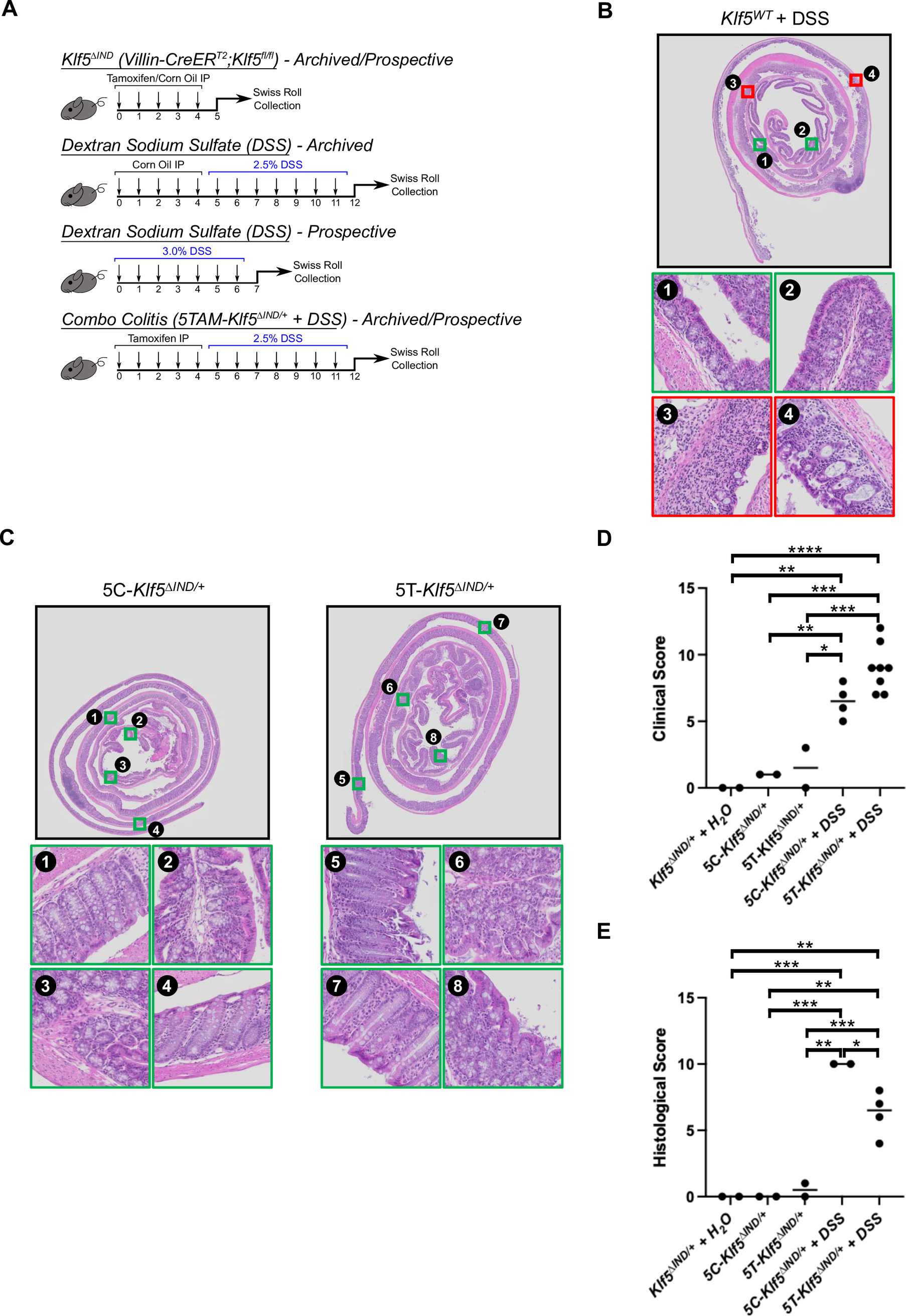

Supplement: S1 Fig — A) Treatment schedules for each mouse model. DSS model in prospective cohort has no injections to confirm abnormal pathology is recognized independent of corn oil. Additionally, 3.0% DSS was used in prospective mice, as this was observed to be the optimal concentration at the Stony Brook facilities. B) Swiss roll of Klf5WT (Villin-CreERT2;Klf5+/+) mouse treated with DSS and no TAM or CO injections. C) Though not used to train our classifier, swiss rolls of corresponding controls (5T-Klf5ΔIND/+ and 5C-Klf5ΔIND/+) are shown for combined colitis model. D) Clinical scores combining weight loss, stool consistency, and fecal blood according to Cooper et al. [17] for combined colitis model, 5C-Klf5ΔIND/+ + DSS, and control mice. E) Histological scores according to Cooper et al. [17]. One-way ANOVA was performed for D) and E). *p<0.0332, **p<0.0021, ***p<0.0002, **** p<0.0001. (TIF) [file pone.0268954.s002.tif]

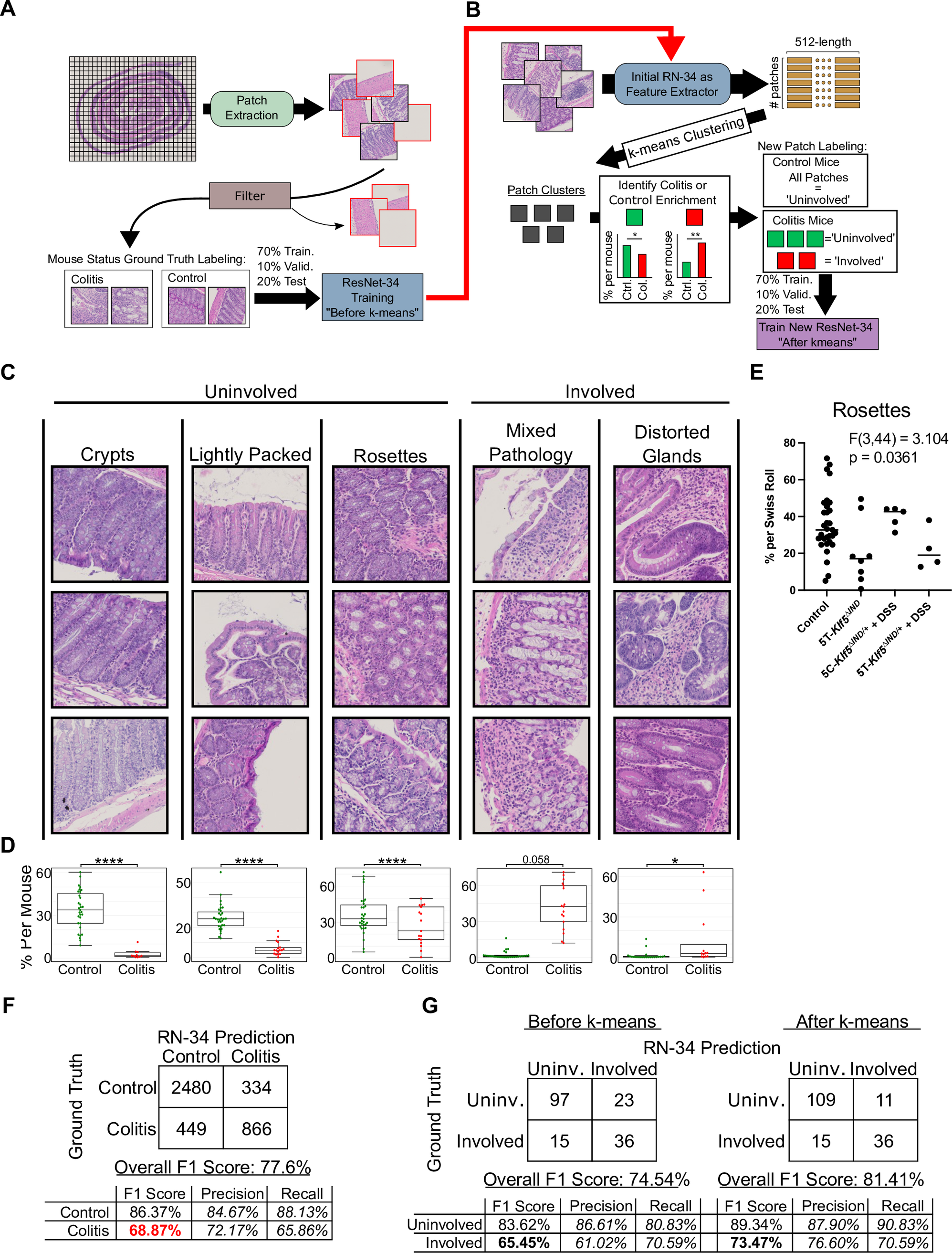

Supplement: S2 Fig — A) Overview schematic showing initial phase of RN-34 model training that uses mouse colitis status as patch ground truth labels. Thus, intracolonic heterogeneity in colitis mice is not addressed at this round of patch labeling. B) Second phase of model training that uses trained RN-34 model from A) as a feature extractor for patches in dataset. K-means clustering on extracted features generated patch classes. Student t-test is utilized to assess whether patch classes are significantly enriched in colitis or control mice. Patches from control mice are labeled as ‘Uninvolved’, as no colitis induction occurred. For colitis mice, patches are labelled ‘Uninvolved’ or ‘Involved’ according to k-means predictions. C) K-means patch classes identified during second phase of training in B) used for ground truth labeling. D) Box and whisker plots of patch class proportions. Lines in center of box indicate median. Box boundaries refer to 1st and 3rd interquartile ranges (IQRs). Whiskers extend to furthest point within to 1.5*IQR. Student’s t-tests were performed. *p<0.05, **p<0.01, ***p<0.001, ****p<0.0001. E) Rosettes are in DSS-treated mice have higher means relative to other colitis models. One-way ANOVA shows a statistically significant difference between groups. F) Independent test set output confusion matrix for initial phase model. G) 200 patches from 4 mice (1 control, 1 of each colitis mouse model) were labeled as ‘Uninvolved’ or ‘Involved’ by a pathologist. 29/200 patches were discarded for not enough spatial context to provide a label. Inference using models trained in A) and B) show that the k-means patch labeling approach increased prediction agreement with pathologist-generated labels. (TIF) [file pone.0268954.s003.tif]

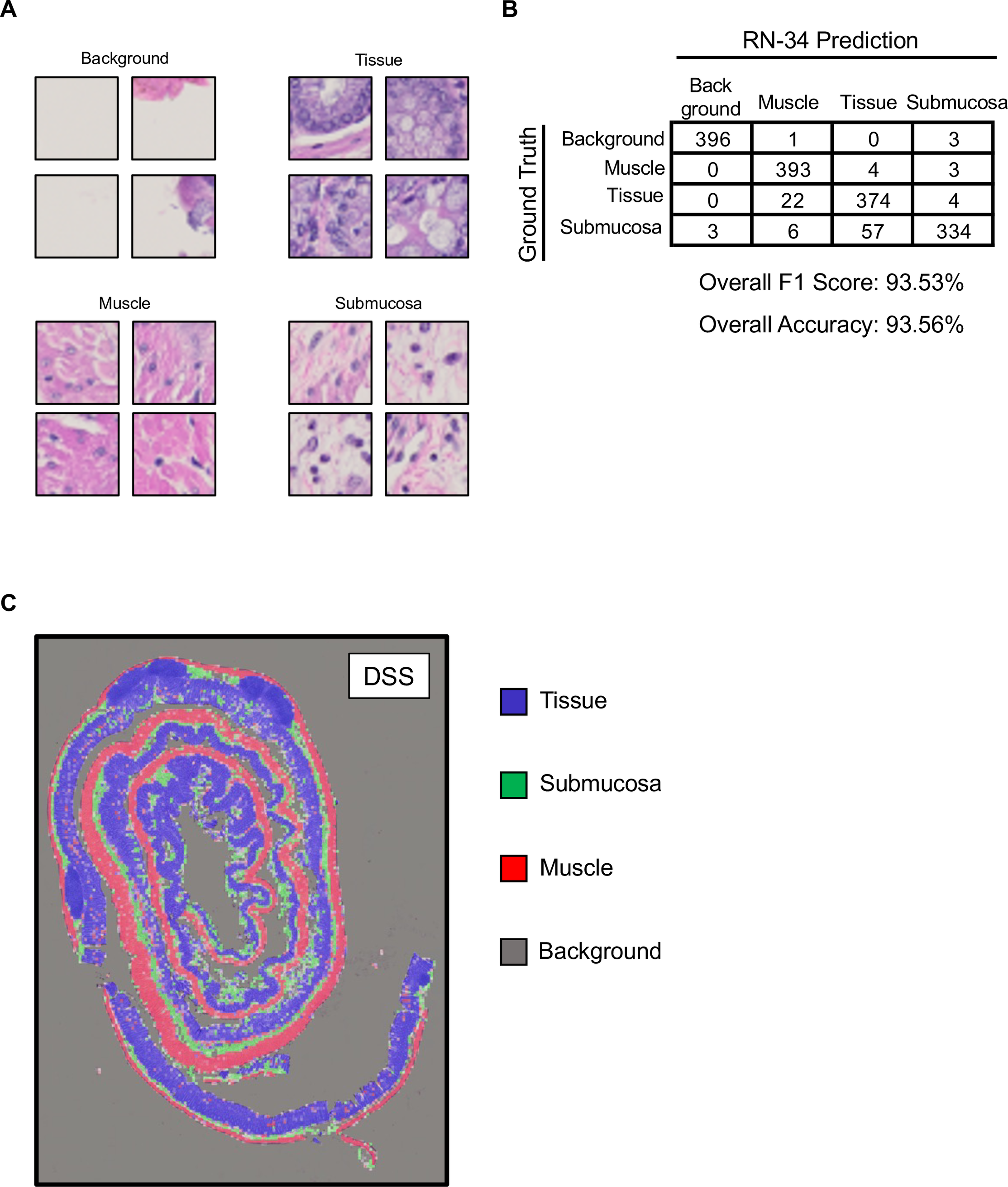

Supplement: S3 Fig — A) Example 32x32 pixel patches for the ‘Background’, ‘Tissue’, ‘Muscle’, and ‘Submucosa’ classes used to train the Small Patch RN-34 Classifier. B) Trained Small Patch Clasifier confusion matrix outputs for independent test set of 4 mice, each with 100 patches of each class (1600 total patches). C) Example overlay of Small Patch Classifier on DSS-treated test set mouse. (TIF) [file pone.0268954.s004.tif]

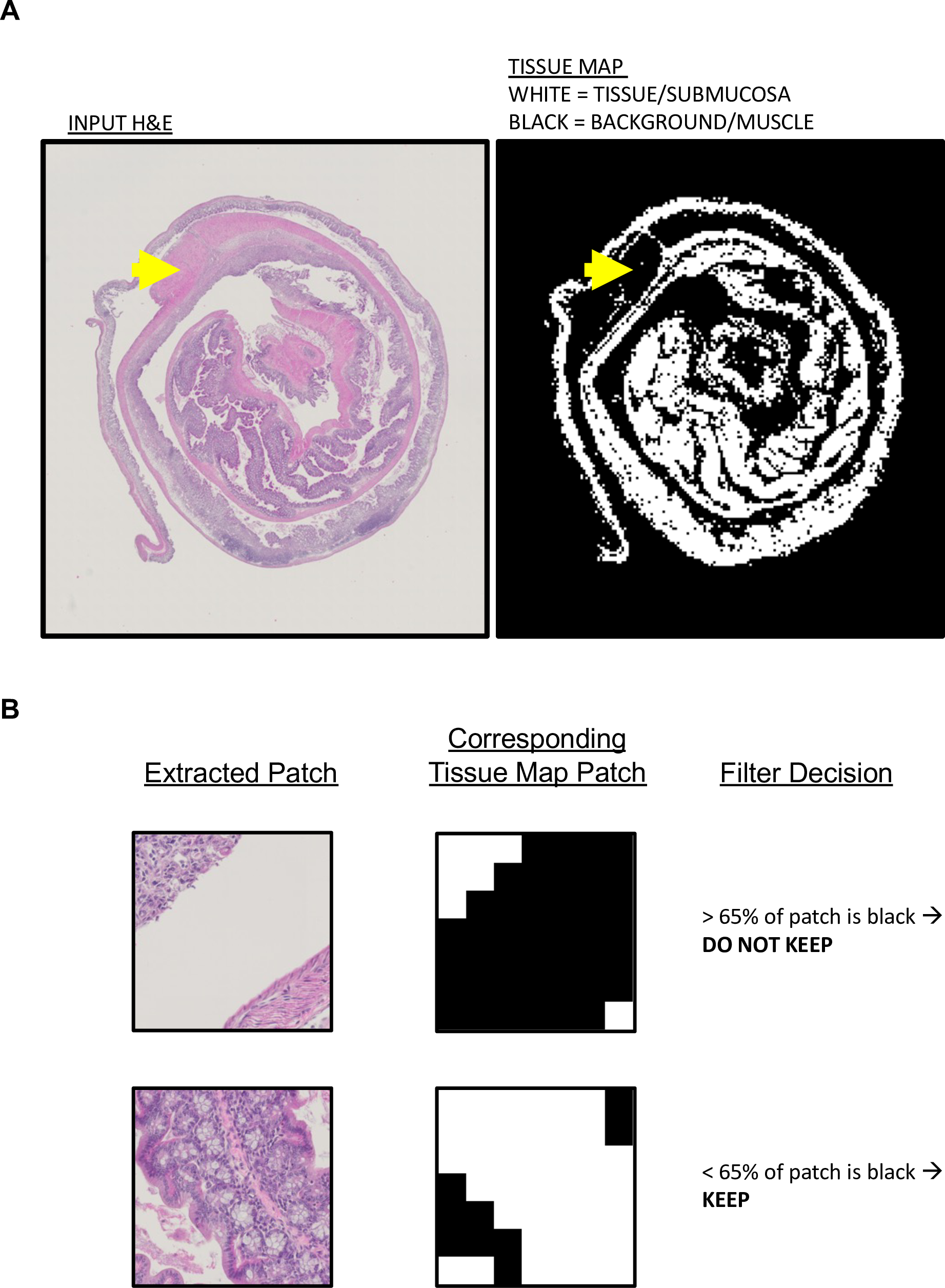

Supplement: S4 Fig — A) Small Patch Classifier from S3 Fig is applied to all 32x32 pixel patches extracted from a WSI. A tissue map is generated where ‘Background’ and ‘Muscle’ are black, while ‘Tissue’ and ‘Submucosa’ are white. The yellow arrow indicates a portion of muscle that is assigned to the Background/Muscle class on the corresponding tissue map. B) Example 224x224 pixel H&E patches with corresponding tissue map patches and filtering decisions. For each extracted patch, the decision is made based on whether there is more than 65% (filter) or less than 65% (keep) of unwanted Background/Muscle area on the corresponding tissue map patch. (TIF) [file pone.0268954.s005.tif]

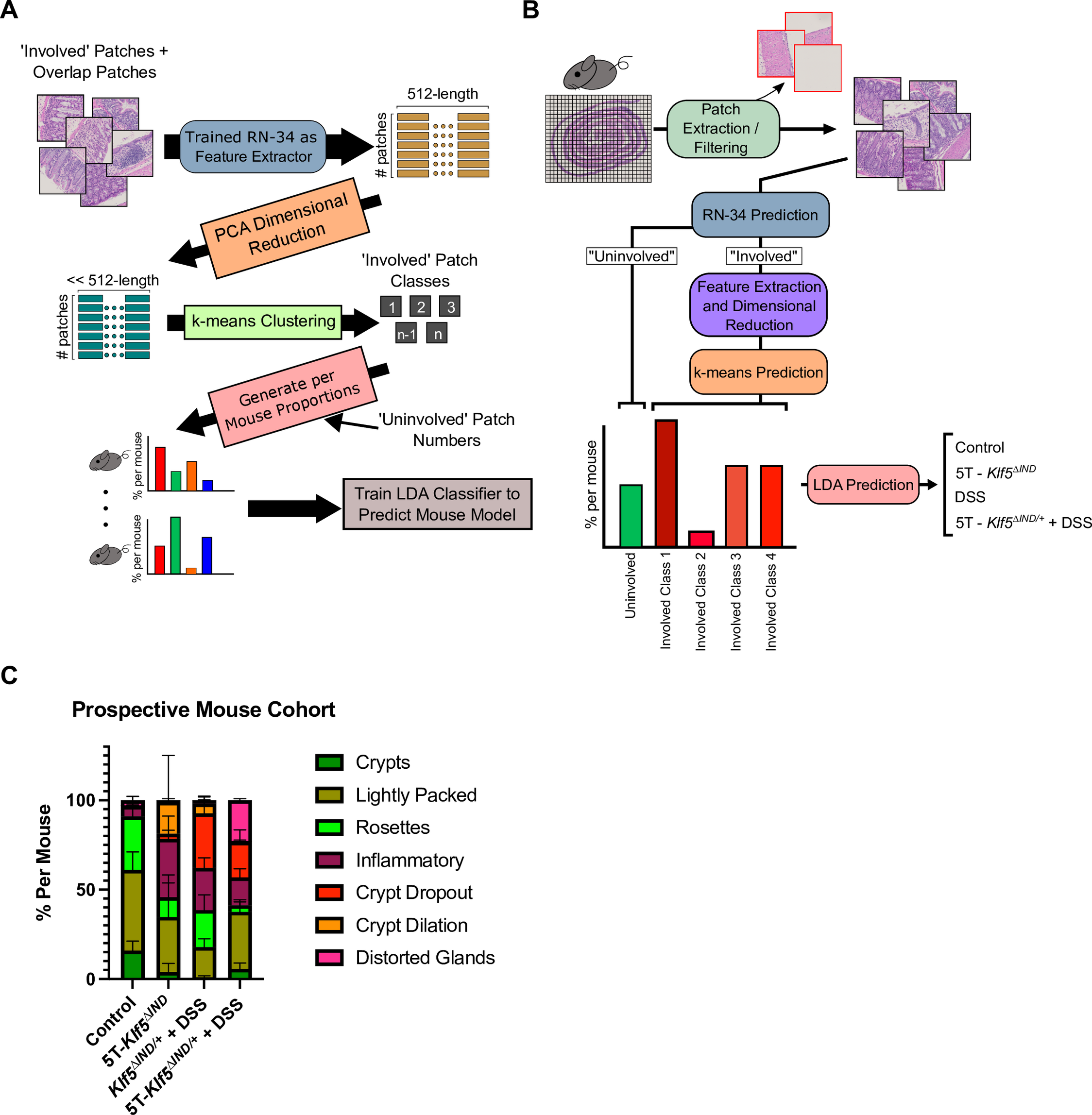

Supplement: S5 Fig — A) Overview schematic. All patches classified as ‘Involved’ by our model, including overlapping patches, undergo feature extraction by our final ‘Involved’ versus ‘Uninvolved’ classifier. Subsequent PCA-based dimensionality reduction and k-means clustering identify 4 ‘Involved’ patch classes (Fig 4A). An LDA classifier is trained on per mice proportions of ‘Uninvolved’ patches and ‘Involved’ k-means patch classes to predict mouse models. B) Overview schematic for inference pipeline. Overlapping patches are extracted and patches containing too much background or muscle are filtered out via the tissue map process in S4 Fig. The classifier is applied to each kept patch. ‘Involved’ patches undergo further RN-34 feature extraction and PCA-based dimensionality reduction. These patches are then classified ‘into one of the four ‘Involved’ patch classes. The trained LDA model then predicts mouse model from the per-mouse proportions of ‘Uninvolved’ patch and ‘Involved’ k-means patch classes. C) Stacked bar plot of total ‘Uninvolved’ and ‘Involved’ k-means patch class proportions across prospective mouse cohort. Error bars show mean with standard deviation. (TIF) [file pone.0268954.s006.tif]

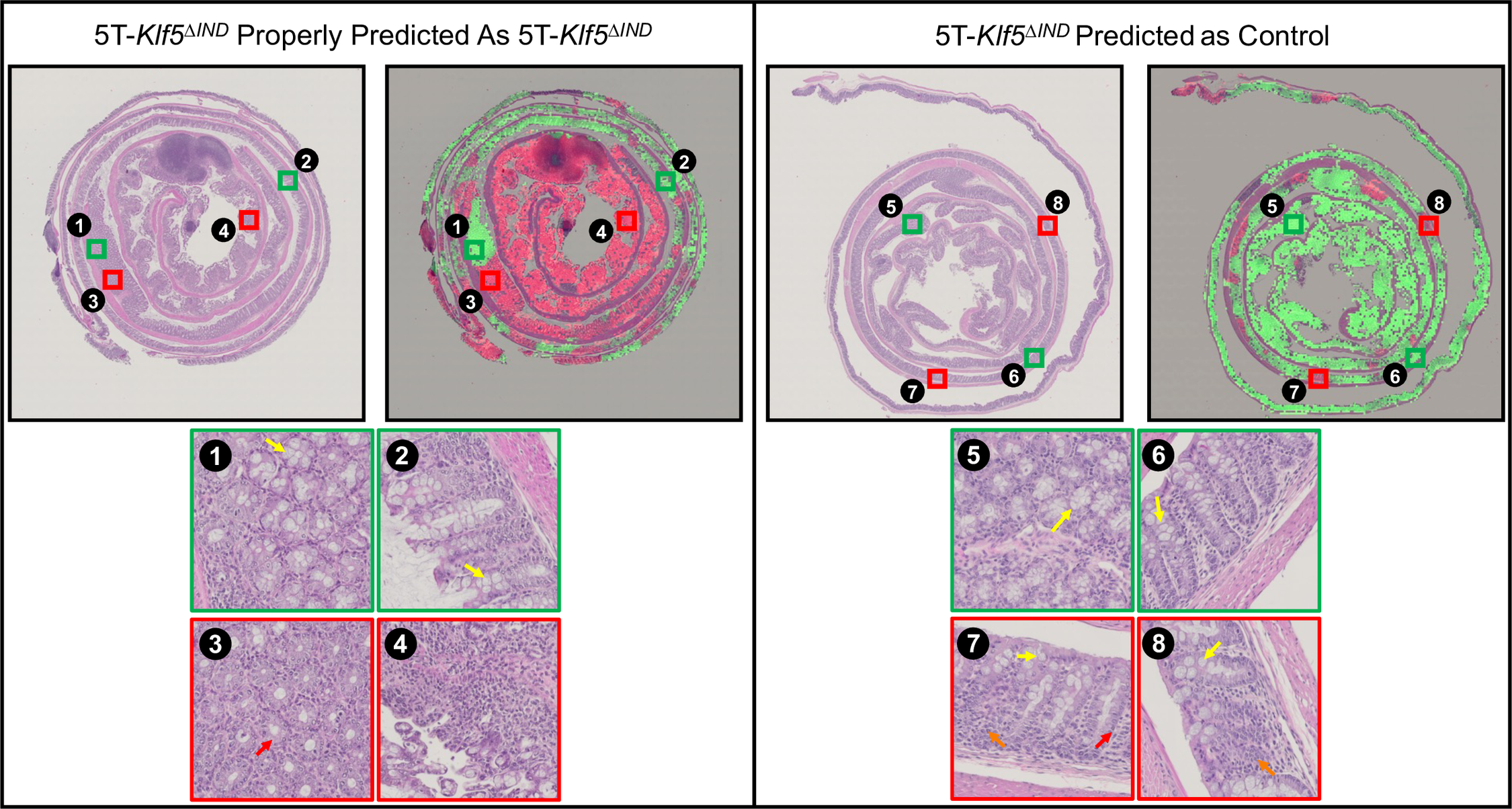

Supplement: S6 Fig — Compared to the properly predicted 5T-Klf5ΔIND swiss rolled colon (left), the 5T-Klf5ΔIND colon predicted as control has fewer histological abnormalities and represents a mouse with weak colitis induction. Yellow arrows indicate healthy goblet cells. Red arrows indicate absence of goblet cells. Orange arrows indicate crypt loss. (TIF) [file pone.0268954.s007.tif]

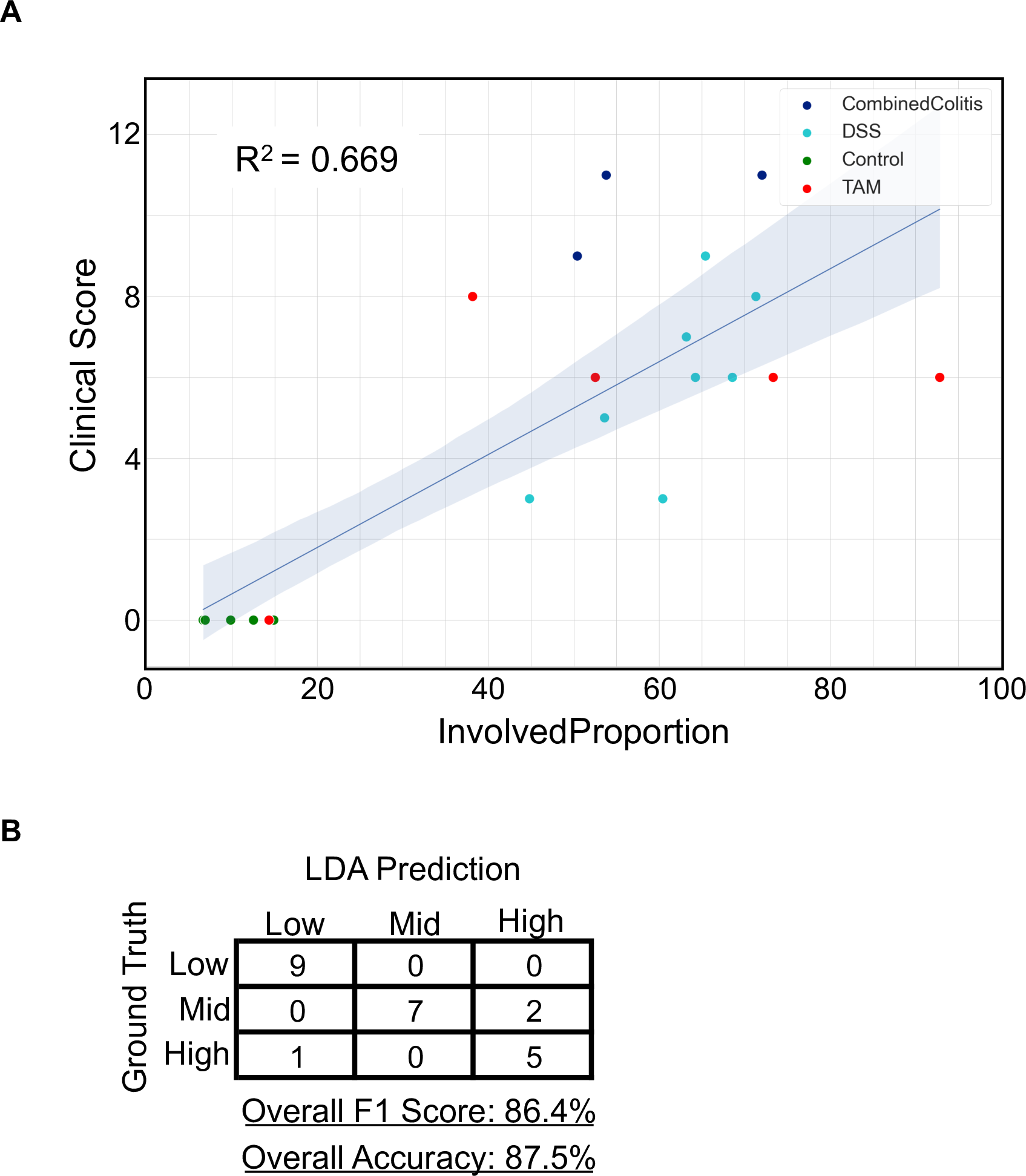

Supplement: S7 Fig — A) Scatter plot of Clinical Score verse InvolvedProportion. Clinical scores were obtained according to Cooper et al. [17]. InvolvedProportion is the proportion of ‘Involved’-predicted pixels out of all prediction pixels in overlays. B) LDA trained on archived mouse cohort predicts prospective mouse cohort clinical score bins from per-mouse ‘Uninvolved’ patch and ‘Involved’ k-means patch class proportions. Clinical score bins are “Low” (0–2), “Mid” (3–7), and “High” (8–12). (TIF) [file pone.0268954.s008.tif]
